# *catena*-Poly[bis(μ_3_-3-aminobenzoato-κ^4^ *N*:*O*:*O*,*O*′)bis(μ_2_-3-aminobenzoato-κ^3^ *O*,*O*′:*O*)dilead(II)]

**DOI:** 10.1107/S1600536810041322

**Published:** 2010-10-23

**Authors:** Fwu Ming Shen, Shie Fu Lush

**Affiliations:** a Department of Biotechnology, Yuanpei University, HsinChu, 30015 Taiwan; b Department of General Education Center, Yuanpei University, No. 306 Yuanpei St, HsinChu, 30015 Taiwan

## Abstract

The Pb^II ^atom in the title compound, {[Pb_2_(C_7_H_6_NO_2_)_4_]}_*n*_, is chelated by two 3-aminobenzoato ligands in a distorted pentagonal-bipyramidal coordination geometry with five oxygen donors in the equatorial positions, one nitro­gen donor and one oxygen donor in the axial positions. Two mol­ecules are linked through a centre of inversion, forming a dinuclear entity. These entities are linked in a μ_3_-bridging mode through the amino N atom and two carboxyl­ate O atoms into a chain along the *b* axis. Classical inter­molecular N—H⋯O hydrogen bonding is observed in the structure. The supra­molecular structure is consolidated by π–π stacking inter­actions with  centroid–centroid distances between benzene rings of 3.837 (8) Å.

## Related literature

For related structures, see: Tan *et al.* (2006 ▶); Wang *et al.* (2004 ▶, 2006 ▶); Wei *et al.* (2006 ▶). 
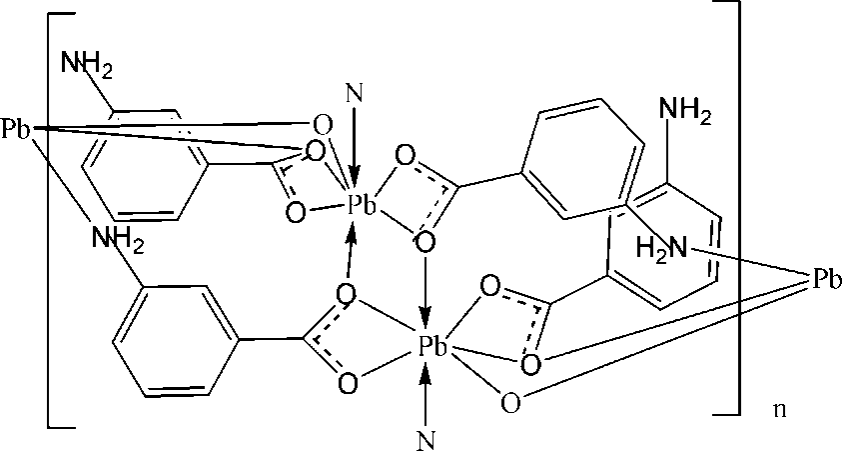


## Experimental

### 

#### Crystal data

[Pb_2_(C_7_H_6_NO_2_)_4_]
*M* *_r_* = 958.92
Triclinic, 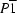

*a* = 6.8610 (3) Å
*b* = 7.8943 (3) Å
*c* = 13.9022 (8) Å
α = 76.030 (2)°
β = 88.103 (2)°
γ = 70.154 (2)°
*V* = 686.33 (6) Å^3^
*Z* = 1
Mo *K*α radiation
μ = 12.31 mm^−1^
*T* = 295 K
0.18 × 0.16 × 0.02 mm

#### Data collection

Nonius KappaCCD diffractometer
Absorption correction: multi-scan (*SCALEPACK*; Otwinowski & Minor, 1997 ▶) *T*_min_ = 0.215, *T*_max_ = 0.791
5451 measured reflections
2470 independent reflections
2218 reflections with *I* > 2σ(*I*)
*R*_int_ = 0.103

#### Refinement

*R*[*F* ^2^ > 2σ(*F* ^2^)] = 0.065
*wR*(*F* ^2^) = 0.169
*S* = 1.05
2470 reflections
190 parameters
12 restraints
H-atom parameters constrained
Δρ_max_ = 3.23 e Å^−3^
Δρ_min_ = −5.12 e Å^−3^

### 

Data collection: *COLLECT* (Nonius, 2000 ▶); cell refinement: *SCALEPACK* (Otwinowski & Minor, 1997 ▶); data reduction: *DENZO* (Otwinowski & Minor, 1997 ▶) and *SCALEPACK*; program(s) used to solve structure: *SHELXS97* (Sheldrick, 2008 ▶); program(s) used to refine structure: *SHELXL97* (Sheldrick, 2008 ▶); molecular graphics: *PLATON* (Spek, 2009 ▶); software used to prepare material for publication: *PLATON*.

## Supplementary Material

Crystal structure: contains datablocks global, I. DOI: 10.1107/S1600536810041322/rk2229sup1.cif

Structure factors: contains datablocks I. DOI: 10.1107/S1600536810041322/rk2229Isup2.hkl

Additional supplementary materials:  crystallographic information; 3D view; checkCIF report

## Figures and Tables

**Table 1:** Hydrogen-bond geometry (Å, °)

*D*—H⋯*A*	*D*—H	H⋯*A*	*D*⋯*A*	*D*—H⋯*A*
N2—H2*A*⋯O4^i^	0.86	2.52	3.037 (12)	119
N2—H2*B*⋯O1^i^	0.86	2.32	2.936 (12)	129

Symmetry code: (i) 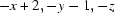.

**Fig. 1.**
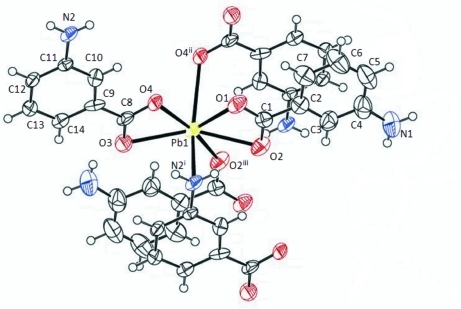
View of the title compound with the atom numbering scheme. Displacement ellipsoids are drawn at the 50% probability level. H atoms are presented as a small spheres of arbitrary radius.

## supplementary crystallographic information

### Comment

In the last few years, much research on metal–organic framework has mostly focused on coordination polymer with rigid organic ligands containing either N– or O–atom donors, such as 3–aminobenzoic acid. Up to now, only few examples of coordination polymer with 3–aminobenzoic acid have been reported (Wang *et al.*, 2004, 2006; Tan *et al.*, 2006; Wei *et al.*, 2006.), and the polymeric complexes of non–transition metal with 3–aminobenzoic acid have not yet been found.

Herein we report the syntheses and crystal structure of Pb(II) coordination polymer with µ3–bridged 3–aminobenzoic acid (Fig.1). The title compound is a one–dimensional coordination polymer based on infinite µ3–bridging chains along *b* axial direction. The Pb···Pb distances are 4.118 (3)Å and 4.491 (3)Å. The Pb^II^ ion is a pentagonal–bipyramidal coordination environment and is coordinated by five carboxyl oxygen atoms comprise the equatorial plane, one nitrogen and one oxygen atom occupies the axial positions. The classic N—H···O hydrogen–bondings are observed in the structure (full details and symmetry codes shown as Table 1).

The supramolecular structure is also consolidated by π—π stacking interaction. The distance between *Cg*1 (C2–C7)···*Cg*2^ii^ (C9–C14) is 3.842 (8)Å and dihedral angle between two benzene rings is 4.6 (7)°. Symmetry code: (ii) 1-*x*, -*y*, -*y*.

### Experimental

A solution of 3-aminobenzoic acid (0.0683 g, 0.50 mmol) in 5 ml deionized water was slowly added to a stirring solution of Pb(NO_3_)_2_^.^6H_2_O (0.1670 g, 0.50 mmol) and 1,2–bis(4–pyridyl)ethane (0.0926 g, 0.50 mmol) in 5 ml deionized water. The mixture solution was seal in 25 ml stainless steel reactor with a teflon liner, heat to 423 K for 24 hr, and then slowly cooled to room temperature. The pink single crystals of the title compound were obtained in 56.45% yield. (Based on Pb).

### Refinement

H atoms were positioned geometrically N—H = 0.86Å and C—H = 0.93Å (aromatic), and were refined using a riding model with *U*_iso_(H) = 1.2*U*_eq_(C,N).

### Crystal data

**Table:**

[Pb_2_(C_7_H_6_NO_2_)_4_]	*Z* = 1
*M**_r_* = 958.92	*F*(000) = 448
Triclinic, *P*1	*D*_x_ = 2.320 Mg m^−^^3^
Hall symbol: -P 1	Mo *K*α radiation, λ = 0.71073 Å
*a* = 6.8610 (3) Å	Cell parameters from 12232 reflections
*b* = 7.8943 (3) Å	θ = 2.0–25.4°
*c* = 13.9022 (8) Å	µ = 12.31 mm^−^^1^
α = 76.030 (2)°	*T* = 295 K
β = 88.103 (2)°	Prism, pink
γ = 70.154 (2)°	0.18 × 0.16 × 0.02 mm
*V* = 686.33 (6) Å^3^	

### Data collection

**Table:**

Nonius KappaCCD diffractometer	2470 independent reflections
Radiation source: fine–focus sealed tube	2218 reflections with *I* > 2σ(*I*)
graphite	*R*_int_ = 0.103
Detector resolution: 9 pixels mm^-1^	θ_max_ = 25.3°, θ_min_ = 2.8°
CCD rotation images, thick slices scans	*h* = −7→8
Absorption correction: multi-scan (*SCALEPACK*; Otwinowski & Minor, 1997)	*k* = −7→9
*T*_min_ = 0.215, *T*_max_ = 0.791	*l* = −16→16
5451 measured reflections	

### Refinement

**Table:**

Refinement on *F*^2^	Primary atom site location: structure-invariant direct methods
Least-squares matrix: full	Secondary atom site location: difference Fourier map
*R*[*F*^2^ > 2σ(*F*^2^)] = 0.065	Hydrogen site location: inferred from neighbouring sites
*wR*(*F*^2^) = 0.169	H-atom parameters constrained
*S* = 1.05	*w* = 1/[σ^2^(*F*_o_^2^) + (0.1239*P*)^2^ + 1.1995*P*] where *P* = (*F*_o_^2^ + 2*F*_c_^2^)/3
2470 reflections	(Δ/σ)_max_ = 0.001
190 parameters	Δρ_max_ = 3.23 e Å^−^^3^
12 restraints	Δρ_min_ = −5.12 e Å^−^^3^

### Special details

**Table:**

Geometry. All s.u.'s (except the s.u. in the dihedral angle between two l.s. planes) are estimated using the full covariance matrix. The cell s.u.'s are taken into account individually in the estimation of s.u.'s in distances, angles and torsion angles; correlations between s.u.'s in cell parameters are only used when they are defined by crystal symmetry. An approximate (isotropic) treatment of cell s.u.'s is used for estimating s.u.'s involving l.s. planes.
Refinement. Refinement of *F*^2^ against ALL reflections. The weighted *R*–factor *wR* and goodness of fit *S* are based on *F*^2^, conventional *R*–factors *R* are based on *F*, with *F* set to zero for negative *F*^2^. The threshold expression of *F*^2^ > σ(*F*^2^) is used only for calculating *R*–factors(gt) *etc*. and is not relevant to the choice of reflections for refinement. *R*–factors based on *F*^2^ are statistically about twice as large as those based on *F*, and *R*–factors based on ALL data will be even larger. The residual peaks show two relatively high peaks of 3.19 eÅ^-3^ at 0.405, 0.645, 0.123 and 3.13 eÅ^-3^ at 0.566, 0.080, 0.038, and the distances to the nearest Pb atoms are 1.26Å and 1.50Å, respectively.

### Fractional atomic coordinates and isotropic or equivalent isotropic displacement parameters (Å^2^)

**Table:**

	*x*	*y*	*z*	*U*_iso_*/*U*_eq_	
Pb1	0.57722 (5)	0.23064 (4)	−0.04961 (3)	0.0375 (1)	
O1	0.7342 (13)	0.0825 (10)	0.1159 (6)	0.051 (3)	
O2	0.6749 (13)	0.3823 (11)	0.0817 (6)	0.051 (3)	
O3	0.6695 (17)	0.0912 (11)	−0.2133 (6)	0.058 (3)	
O4	0.7229 (12)	−0.0859 (9)	−0.0620 (5)	0.040 (2)	
N1	0.655 (3)	0.555 (2)	0.4054 (11)	0.092 (6)	
N2	0.9270 (14)	−0.7459 (12)	−0.0976 (7)	0.038 (3)	
C1	0.7141 (15)	0.2286 (15)	0.1435 (8)	0.037 (3)	
C2	0.7253 (16)	0.2209 (16)	0.2521 (9)	0.043 (3)	
C3	0.6951 (19)	0.3817 (19)	0.2811 (9)	0.054 (4)	
C4	0.696 (2)	0.383 (2)	0.3805 (10)	0.065 (4)	
C5	0.733 (2)	0.212 (3)	0.4500 (10)	0.076 (5)	
C6	0.768 (3)	0.050 (3)	0.4203 (11)	0.079 (5)	
C7	0.766 (2)	0.056 (2)	0.3199 (10)	0.057 (4)	
C8	0.7220 (17)	−0.0662 (14)	−0.1556 (8)	0.039 (3)	
C9	0.7796 (14)	−0.2349 (13)	−0.1927 (8)	0.035 (3)	
C10	0.8350 (14)	−0.4092 (14)	−0.1314 (8)	0.035 (3)	
C11	0.8749 (15)	−0.5654 (13)	−0.1674 (7)	0.029 (3)	
C12	0.8668 (17)	−0.5505 (15)	−0.2690 (8)	0.037 (3)	
C13	0.8113 (19)	−0.3757 (16)	−0.3344 (8)	0.043 (3)	
C14	0.7646 (16)	−0.2166 (14)	−0.2971 (8)	0.040 (3)	
H1A	0.63110	0.65490	0.35920	0.1100*	
H1B	0.65500	0.55860	0.46660	0.1100*	
H2A	0.93270	−0.75440	−0.03490	0.0460*	
H2B	0.95220	−0.84430	−0.11910	0.0460*	
H3	0.67370	0.49200	0.23310	0.0660*	
H5	0.73270	0.20860	0.51740	0.0920*	
H6	0.79390	−0.06200	0.46730	0.0940*	
H7	0.79250	−0.05300	0.29910	0.0690*	
H10	0.84610	−0.42310	−0.06320	0.0420*	
H12	0.89830	−0.65640	−0.29310	0.0450*	
H13	0.80500	−0.36400	−0.40250	0.0510*	
H14	0.72400	−0.09940	−0.34050	0.0480*	

### Atomic displacement parameters (Å^2^)

**Table:**

	*U*^11^	*U*^22^	*U*^33^	*U*^12^	*U*^13^	*U*^23^
Pb1	0.0414 (2)	0.0231 (2)	0.0487 (3)	−0.0120 (2)	0.0023 (2)	−0.0088 (2)
O1	0.056 (5)	0.034 (4)	0.065 (5)	−0.015 (4)	0.007 (4)	−0.019 (4)
O2	0.061 (5)	0.035 (4)	0.059 (5)	−0.020 (4)	−0.004 (4)	−0.010 (4)
O3	0.094 (6)	0.034 (4)	0.051 (5)	−0.028 (4)	0.010 (4)	−0.013 (4)
O4	0.058 (5)	0.023 (3)	0.043 (4)	−0.013 (3)	0.007 (3)	−0.015 (3)
N1	0.113 (11)	0.118 (11)	0.067 (7)	−0.049 (10)	0.027 (7)	−0.054 (8)
N2	0.046 (5)	0.026 (4)	0.048 (5)	−0.016 (4)	0.005 (4)	−0.014 (4)
C1	0.027 (4)	0.038 (5)	0.046 (5)	−0.009 (4)	0.010 (4)	−0.014 (4)
C2	0.029 (5)	0.046 (6)	0.051 (6)	−0.009 (4)	0.004 (4)	−0.015 (4)
C3	0.050 (6)	0.063 (7)	0.055 (6)	−0.022 (6)	−0.003 (5)	−0.019 (6)
C4	0.044 (7)	0.091 (8)	0.066 (7)	−0.015 (7)	0.012 (6)	−0.041 (6)
C5	0.065 (8)	0.123 (11)	0.044 (6)	−0.032 (9)	0.013 (6)	−0.027 (6)
C6	0.081 (10)	0.080 (9)	0.053 (7)	−0.010 (9)	0.003 (7)	−0.001 (7)
C7	0.048 (7)	0.053 (7)	0.059 (7)	−0.008 (6)	0.008 (6)	−0.006 (6)
C8	0.046 (5)	0.023 (5)	0.053 (6)	−0.018 (4)	0.006 (5)	−0.012 (4)
C9	0.025 (4)	0.022 (4)	0.056 (6)	−0.011 (4)	0.007 (4)	0.000 (4)
C10	0.021 (4)	0.030 (5)	0.049 (5)	0.003 (4)	−0.002 (4)	−0.018 (4)
C11	0.027 (4)	0.024 (4)	0.042 (5)	−0.013 (4)	0.007 (4)	−0.013 (4)
C12	0.048 (6)	0.036 (5)	0.041 (5)	−0.024 (4)	0.012 (4)	−0.021 (4)
C13	0.058 (6)	0.040 (6)	0.038 (5)	−0.024 (5)	0.005 (5)	−0.014 (4)
C14	0.044 (5)	0.029 (5)	0.050 (6)	−0.016 (4)	0.003 (5)	−0.010 (4)

### Geometric parameters (Å, °)

**Table:**

Pb1—O1	2.415 (8)	C3—C4	1.385 (18)
Pb1—O2	2.626 (8)	C4—C5	1.41 (2)
Pb1—O3	2.726 (8)	C5—C6	1.38 (3)
Pb1—O4	2.403 (7)	C6—C7	1.39 (2)
Pb1—N2^i^	2.525 (10)	C8—C9	1.469 (15)
Pb1—O4^ii^	2.913 (8)	C9—C10	1.365 (15)
Pb1—O2^iii^	2.887 (8)	C9—C14	1.427 (15)
O1—C1	1.264 (14)	C10—C11	1.380 (15)
O2—C1	1.254 (14)	C11—C12	1.390 (14)
O3—C8	1.246 (14)	C12—C13	1.392 (16)
O4—C8	1.273 (13)	C13—C14	1.407 (16)
N1—C4	1.42 (2)	C3—H3	0.9300
N2—C11	1.452 (13)	C5—H5	0.9300
N1—H1B	0.8600	C6—H6	0.9300
N1—H1A	0.8600	C7—H7	0.9300
N2—H2B	0.8600	C10—H10	0.9300
N2—H2A	0.8600	C12—H12	0.9300
C1—C2	1.500 (16)	C13—H13	0.9300
C2—C7	1.356 (19)	C14—H14	0.9300
C2—C3	1.371 (19)		
			
Pb1···C3^iii^	3.870 (13)	C3···C13^ii^	3.542 (19)
Pb1···C10^ii^	3.854 (11)	C3···C14^vi^	3.482 (18)
Pb1···H3^iii^	3.0500	C4···C13^vi^	3.40 (2)
Pb1···H10^ii^	3.3300	C4···C13^ii^	3.58 (2)
O1···O2	2.193 (11)	C7···C14^ii^	3.422 (19)
O1···O4	3.092 (11)	C8···Pb1^ii^	3.698 (11)
O1···C2	2.394 (15)	C9···C1^ii^	3.419 (15)
O1···N2^iv^	2.936 (12)	C9···C1^vi^	3.582 (15)
O1···C8^ii^	3.200 (15)	C9···C2^vi^	3.501 (16)
O1···O4^ii^	3.238 (13)	C9···C2^ii^	3.547 (16)
O2···N2^i^	3.158 (13)	C10···O2^v^	3.369 (14)
O2···C2	2.378 (15)	C10···Pb1^ii^	3.854 (11)
O2···O1	2.193 (11)	C10···C1^ii^	3.540 (15)
O2···O2^iii^	3.109 (12)	C13···C3^ii^	3.542 (19)
O2···C10^i^	3.369 (14)	C13···C3^vi^	3.473 (19)
O3···C9	2.370 (13)	C13···C4^vi^	3.40 (2)
O3···O4	2.190 (11)	C13···C4^ii^	3.58 (2)
O3···N2^i^	3.177 (14)	C14···C3^vi^	3.482 (18)
O4···O1	3.092 (11)	C14···C2^vi^	3.563 (17)
O4···C9	2.348 (12)	C14···C2^ii^	3.409 (17)
O4···O1^ii^	3.238 (13)	C14···C7^ii^	3.422 (19)
O4···N2^iv^	3.037 (12)	C1···H2A^i^	2.8500
O4···O3	2.190 (11)	C5···H1B^vii^	3.0400
O1···H7	2.5000	C8···H2B^i^	2.8600
O1···H2B^iv^	2.3200	H1A···H3	2.3700
O1···H2A^i^	2.7600	H1A···O3^iii^	2.8100
O2···H10^i^	2.7200	H1B···H5	2.5500
O2···H2A^i^	2.5100	H1B···H13^viii^	2.4100
O2···H3	2.4600	H1B···C5^vii^	3.0400
O3···H2B^i^	2.6300	H2A···H10	2.4100
O3···H1A^iii^	2.8100	H2A···O4^iv^	2.5200
O3···H14	2.5300	H2B···H12	2.4800
O4···H10	2.5100	H2B···O1^iv^	2.3200
O4···H2B^i^	2.8200	H3···O2	2.4600
O4···H2A^iv^	2.5200	H3···H1A	2.3700
N2···O2^v^	3.158 (13)	H3···Pb1^iii^	3.0500
N2···O3^v^	3.177 (14)	H5···H1B	2.5500
N2···O1^iv^	2.936 (12)	H7···O1	2.5000
N2···O4^iv^	3.037 (12)	H7···H12^iv^	2.5600
C1···C10^ii^	3.540 (15)	H10···O2^v^	2.7200
C1···C9^ii^	3.419 (15)	H10···O4	2.5100
C1···C9^vi^	3.582 (15)	H10···H2A	2.4100
C2···C14^vi^	3.563 (17)	H10···Pb1^ii^	3.3300
C2···C9^ii^	3.547 (16)	H10···H10^iv^	2.5500
C2···C9^vi^	3.501 (16)	H12···H2B	2.4800
C2···C14^ii^	3.409 (17)	H12···H7^iv^	2.5600
C3···C13^vi^	3.473 (19)	H13···H1B^ix^	2.4100
C3···Pb1^iii^	3.870 (13)	H14···O3	2.5300
			
O1—Pb1—O2	51.4 (3)	H2A—N2—H2B	120.00
O1—Pb1—O3	126.6 (3)	Pb1^v^—N2—H2B	88.00
O1—Pb1—O4	79.9 (2)	Pb1—C1—O1	56.6 (6)
O1—Pb1—C1	25.9 (3)	O1—C1—O2	121.1 (10)
O1—Pb1—C8	103.8 (3)	Pb1—C1—O2	66.2 (6)
O1—Pb1—N2^i^	86.3 (3)	Pb1—C1—C2	163.1 (8)
O1—Pb1—O4^ii^	74.2 (3)	O1—C1—C2	119.7 (10)
O1—Pb1—O2^iii^	115.3 (2)	O2—C1—C2	119.1 (10)
O2—Pb1—O3	149.9 (3)	C3—C2—C7	121.1 (12)
O2—Pb1—O4	128.1 (3)	C1—C2—C3	119.1 (11)
O2—Pb1—C1	25.9 (3)	C1—C2—C7	119.8 (11)
O2—Pb1—C8	145.9 (3)	C2—C3—C4	121.3 (13)
O2—Pb1—N2^i^	75.6 (3)	N1—C4—C5	124.5 (14)
O2—Pb1—O4^ii^	98.7 (2)	C3—C4—C5	117.1 (14)
O2—Pb1—O2^iii^	68.5 (3)	N1—C4—C3	118.4 (13)
O3—Pb1—O4	50.1 (2)	C4—C5—C6	121.4 (13)
O3—Pb1—C1	147.2 (3)	C5—C6—C7	119.4 (16)
O3—Pb1—C8	25.0 (3)	C2—C7—C6	119.8 (15)
O3—Pb1—N2^i^	74.3 (3)	Pb1—C8—O3	67.9 (6)
O3—Pb1—O4^ii^	109.5 (3)	Pb1—C8—O4	53.2 (5)
O2^iii^—Pb1—O3	117.1 (2)	Pb1—C8—C9	169.2 (8)
O4—Pb1—C1	105.3 (3)	O3—C8—O4	120.9 (10)
O4—Pb1—C8	25.1 (3)	O3—C8—C9	121.5 (10)
O4—Pb1—N2^i^	85.9 (3)	O4—C8—C9	117.7 (9)
O4—Pb1—O4^ii^	81.3 (2)	C8—C9—C10	122.9 (10)
O2^iii^—Pb1—O4	163.4 (2)	C8—C9—C14	118.9 (9)
C1—Pb1—C8	128.2 (3)	C10—C9—C14	118.1 (9)
N2^i^—Pb1—C1	83.5 (3)	C9—C10—C11	122.1 (10)
O4^ii^—Pb1—C1	83.0 (3)	N2—C11—C12	120.4 (9)
O2^iii^—Pb1—C1	90.5 (3)	C10—C11—C12	120.5 (10)
N2^i^—Pb1—C8	80.2 (3)	N2—C11—C10	119.0 (9)
O4^ii^—Pb1—C8	94.8 (3)	C11—C12—C13	119.4 (10)
O2^iii^—Pb1—C8	140.9 (3)	C12—C13—C14	119.8 (10)
O4^ii^—Pb1—N2^i^	158.2 (3)	C9—C14—C13	120.1 (10)
O2^iii^—Pb1—N2^i^	101.2 (3)	C2—C3—H3	119.00
O2^iii^—Pb1—O4^ii^	96.0 (2)	C4—C3—H3	119.00
Pb1—O1—C1	97.5 (7)	C4—C5—H5	119.00
Pb1—O2—C1	87.9 (7)	C6—C5—H5	120.00
Pb1—O2—Pb1^iii^	111.5 (3)	C5—C6—H6	120.00
Pb1^iii^—O2—C1	142.0 (7)	C7—C6—H6	120.00
Pb1—O3—C8	87.1 (6)	C2—C7—H7	120.00
Pb1—O4—C8	101.8 (6)	C6—C7—H7	120.00
Pb1—O4—Pb1^ii^	98.7 (3)	C9—C10—H10	119.00
Pb1^ii^—O4—C8	118.8 (7)	C11—C10—H10	119.00
Pb1^v^—N2—C11	103.4 (7)	C11—C12—H12	120.00
H1A—N1—H1B	120.00	C13—C12—H12	120.00
C4—N1—H1A	120.00	C12—C13—H13	120.00
C4—N1—H1B	120.00	C14—C13—H13	120.00
Pb1^v^—N2—H2A	78.00	C9—C14—H14	120.00
C11—N2—H2A	120.00	C13—C14—H14	120.00
C11—N2—H2B	120.00		
			
O2—Pb1—O1—C1	8.3 (6)	O2—Pb1—N2^i^—C11^i^	−90.7 (6)
O3—Pb1—O1—C1	150.1 (7)	O3—Pb1—N2^i^—C11^i^	88.7 (6)
O4—Pb1—O1—C1	169.3 (7)	O4—Pb1—N2^i^—C11^i^	138.3 (6)
C8—Pb1—O1—C1	161.8 (7)	C1—Pb1—N2^i^—C11^i^	−115.8 (6)
N2^i^—Pb1—O1—C1	82.8 (7)	C8—Pb1—N2^i^—C11^i^	113.6 (6)
O4^ii^—Pb1—O1—C1	−107.0 (7)	O1—Pb1—O4^ii^—Pb1^ii^	−81.9 (3)
O2^iii^—Pb1—O1—C1	−17.8 (8)	O1—Pb1—O4^ii^—C8^ii^	26.8 (7)
O1—Pb1—O2—C1	−8.3 (6)	O2—Pb1—O4^ii^—Pb1^ii^	−127.4 (3)
O1—Pb1—O2—Pb1^iii^	−154.7 (5)	O2—Pb1—O4^ii^—C8^ii^	−18.8 (8)
O3—Pb1—O2—C1	−106.4 (8)	O3—Pb1—O4^ii^—Pb1^ii^	42.0 (3)
O3—Pb1—O2—Pb1^iii^	107.2 (5)	O3—Pb1—O4^ii^—C8^ii^	150.6 (7)
O4—Pb1—O2—C1	−32.3 (8)	O4—Pb1—O4^ii^—Pb1^ii^	0.0 (2)
O4—Pb1—O2—Pb1^iii^	−178.7 (3)	O4—Pb1—O4^ii^—C8^ii^	108.6 (7)
C1—Pb1—O2—Pb1^iii^	−146.4 (8)	C1—Pb1—O4^ii^—Pb1^ii^	−106.8 (3)
C8—Pb1—O2—C1	−58.8 (9)	C1—Pb1—O4^ii^—C8^ii^	1.9 (7)
C8—Pb1—O2—Pb1^iii^	154.7 (4)	C8—Pb1—O4^ii^—Pb1^ii^	21.2 (3)
N2^i^—Pb1—O2—C1	−105.1 (7)	C8—Pb1—O4^ii^—C8^ii^	129.8 (7)
N2^i^—Pb1—O2—Pb1^iii^	108.5 (4)	O1—Pb1—O2^iii^—Pb1^iii^	21.7 (4)
O4^ii^—Pb1—O2—C1	53.4 (7)	O1—Pb1—O2^iii^—C1^iii^	137.8 (12)
O4^ii^—Pb1—O2—Pb1^iii^	−93.1 (3)	O2—Pb1—O2^iii^—Pb1^iii^	0.0 (3)
O2^iii^—Pb1—O2—C1	146.4 (7)	O2—Pb1—O2^iii^—C1^iii^	116.1 (13)
O2^iii^—Pb1—O2—Pb1^iii^	0.0 (3)	O3—Pb1—O2^iii^—Pb1^iii^	−147.5 (3)
O1—Pb1—O3—C8	27.6 (9)	O3—Pb1—O2^iii^—C1^iii^	−31.4 (13)
O2—Pb1—O3—C8	101.9 (9)	C1—Pb1—O2^iii^—Pb1^iii^	14.0 (4)
O4—Pb1—O3—C8	2.7 (7)	C1—Pb1—O2^iii^—C1^iii^	130.1 (12)
C1—Pb1—O3—C8	51.3 (11)	C8—Pb1—O2^iii^—Pb1^iii^	−157.7 (4)
N2^i^—Pb1—O3—C8	100.6 (8)	C8—Pb1—O2^iii^—C1^iii^	−41.6 (14)
O4^ii^—Pb1—O3—C8	−56.9 (8)	Pb1—O1—C1—O2	−16.0 (12)
O2^iii^—Pb1—O3—C8	−164.6 (7)	Pb1—O1—C1—C2	160.6 (9)
O1—Pb1—O4—C8	−162.6 (8)	Pb1—O2—C1—O1	14.5 (11)
O1—Pb1—O4—Pb1^ii^	75.4 (3)	Pb1—O2—C1—C2	−162.0 (9)
O2—Pb1—O4—C8	−143.8 (7)	Pb1^iii^—O2—C1—Pb1	123.3 (11)
O2—Pb1—O4—Pb1^ii^	94.2 (3)	Pb1^iii^—O2—C1—O1	137.8 (10)
O3—Pb1—O4—C8	−2.7 (7)	Pb1^iii^—O2—C1—C2	−38.8 (18)
O3—Pb1—O4—Pb1^ii^	−124.7 (5)	Pb1—O3—C8—O4	−4.6 (12)
C1—Pb1—O4—C8	−157.8 (7)	Pb1—O3—C8—C9	173.8 (11)
C1—Pb1—O4—Pb1^ii^	80.2 (3)	Pb1—O4—C8—O3	5.3 (14)
C8—Pb1—O4—Pb1^ii^	−122.0 (8)	Pb1—O4—C8—C9	−173.1 (8)
N2^i^—Pb1—O4—C8	−75.7 (7)	Pb1^ii^—O4—C8—Pb1	106.9 (6)
N2^i^—Pb1—O4—Pb1^ii^	162.3 (3)	Pb1^ii^—O4—C8—O3	112.2 (12)
O4^ii^—Pb1—O4—C8	122.0 (7)	Pb1^ii^—O4—C8—C9	−66.2 (12)
O4^ii^—Pb1—O4—Pb1^ii^	0.0 (2)	Pb1^v^—N2—C11—C12	−96.2 (10)
O1—Pb1—C1—O2	165.1 (11)	Pb1^v^—N2—C11—C10	84.2 (10)
O2—Pb1—C1—O1	−165.1 (11)	O1—C1—C2—C7	2.9 (17)
O3—Pb1—C1—O1	−47.5 (10)	O1—C1—C2—C3	−177.5 (12)
O3—Pb1—C1—O2	117.6 (7)	O2—C1—C2—C7	179.5 (12)
O4—Pb1—C1—O1	−11.0 (7)	O2—C1—C2—C3	−0.9 (17)
O4—Pb1—C1—O2	154.1 (6)	C7—C2—C3—C4	−3(2)
C8—Pb1—C1—O1	−22.7 (8)	C1—C2—C3—C4	177.5 (12)
C8—Pb1—C1—O2	142.4 (6)	C1—C2—C7—C6	−177.4 (15)
N2^i^—Pb1—C1—O1	−94.8 (7)	C3—C2—C7—C6	3(2)
N2^i^—Pb1—C1—O2	70.3 (7)	C2—C3—C4—N1	−177.3 (15)
O4^ii^—Pb1—C1—O1	68.0 (7)	C2—C3—C4—C5	1(2)
O4^ii^—Pb1—C1—O2	−126.9 (7)	N1—C4—C5—C6	178.7 (18)
O2^iii^—Pb1—C1—O1	163.9 (7)	C3—C4—C5—C6	0(2)
O2^iii^—Pb1—C1—O2	−31.0 (7)	C4—C5—C6—C7	0(3)
O1—Pb1—C8—O3	−157.5 (8)	C5—C6—C7—C2	−2(3)
O1—Pb1—C8—O4	17.6 (8)	O4—C8—C9—C10	−0.6 (17)
O2—Pb1—C8—O3	−119.1 (8)	O3—C8—C9—C10	−179.1 (12)
O2—Pb1—C8—O4	56.0 (10)	O3—C8—C9—C14	−3.2 (17)
O3—Pb1—C8—O4	175.1 (13)	O4—C8—C9—C14	175.2 (10)
O4—Pb1—C8—O3	−175.1 (13)	C14—C9—C10—C11	−0.4 (16)
C1—Pb1—C8—O3	−147.5 (8)	C8—C9—C14—C13	−177.6 (11)
C1—Pb1—C8—O4	27.6 (9)	C8—C9—C10—C11	175.5 (10)
N2^i^—Pb1—C8—O3	−73.8 (8)	C10—C9—C14—C13	−1.6 (16)
N2^i^—Pb1—C8—O4	101.3 (7)	C9—C10—C11—C12	2.3 (17)
O4^ii^—Pb1—C8—O3	127.6 (8)	C9—C10—C11—N2	−178.2 (10)
O4^ii^—Pb1—C8—O4	−57.3 (7)	N2—C11—C12—C13	178.4 (11)
O2^iii^—Pb1—C8—O3	22.0 (10)	C10—C11—C12—C13	−2.1 (18)
O2^iii^—Pb1—C8—O4	−162.9 (6)	C11—C12—C13—C14	0.1 (19)
O1—Pb1—N2^i^—C11^i^	−141.7 (6)	C12—C13—C14—C9	1.7 (18)

Symmetry codes: (i) *x*, *y*+1, *z*; (ii) −*x*+1, −*y*, −*z*; (iii) −*x*+1, −*y*+1, −*z*; (iv) −*x*+2, −*y*−1, −*z*; (v) *x*, *y*−1, *z*; (vi) −*x*+2, −*y*, −*z*; (vii) −*x*+1, −*y*+1, −*z*+1; (viii) *x*, *y*+1, *z*+1; (ix) *x*, *y*−1, *z*−1.

### Hydrogen-bond geometry (Å, °)

**Table:**

*D*—H···*A*	*D*—H	H···*A*	*D*···*A*	*D*—H···*A*
N2—H2A···O4^iv^	0.86	2.52	3.037 (12)	119
N2—H2B···O1^iv^	0.86	2.32	2.936 (12)	129

Symmetry codes: (iv) −*x*+2, −*y*−1, −*z*.
